# Forensic analysis of the microbiome of phones and shoes

**DOI:** 10.1186/s40168-015-0082-9

**Published:** 2015-05-12

**Authors:** Simon Lax, Jarrad T Hampton-Marcell, Sean M Gibbons, Geórgia Barguil Colares, Daniel Smith, Jonathan A Eisen, Jack A Gilbert

**Affiliations:** Institute for Genomics and Systems Biology, Biosciences Department, Argonne National Laboratory, 9700 South Cass Avenue, Argonne, IL 60439 USA; Department of Ecology and Evolution, University of Chicago, 1101 E 57th Street, Chicago, IL 60637 USA; Graduate Program in Biophysical Sciences, University of Chicago, 5801 South Ellis Avenue, Chicago, USA; Departamento de Biologia, Universidade Federal do Ceará, Avenida da Universidade, 2853 - Benfica, Fortaleza, CE 60440-900 Brazil; Alkek Center for Metagenomics and Microbiome Research, Department of Molecular Virology and Microbiology, Baylor College of Medicine, 1 Baylor Plaza, Houston, TX 77030 USA; Department of Evolution and Ecology, University of California, 1544 Newton Ct, Davis, CA USA; Department of Medical Microbiology and Immunology, University of California, 1544 Newton Ct, Davis, CA USA; UC Davis Genome Center, University of California, 1 Shields Avenue, Davis, CA USA; Marine Biological Laboratory, 7 MBL Street, Woods Hole, MA 02543 USA; College of Environmental and Resource Sciences, Zhejiang University, 38 Zheda Road, Hangzhou, 310058 China

**Keywords:** Forensic microbiology, Source-sink dynamics, Shoe microbiome, Phone microbiome, Microbial time series

## Abstract

**Background:**

Microbial interaction between human-associated objects and the environments we inhabit may have forensic implications, and the extent to which microbes are shared between individuals inhabiting the same space may be relevant to human health and disease transmission. In this study, two participants sampled the front and back of their cell phones, four different locations on the soles of their shoes, and the floor beneath them every waking hour over a 2-day period. A further 89 participants took individual samples of their shoes and phones at three different scientific conferences.

**Results:**

Samples taken from different surface types maintained significantly different microbial community structures. The impact of the floor microbial community on that of the shoe environments was strong and immediate, as evidenced by Procrustes analysis of shoe replicates and significant correlation between shoe and floor samples taken at the same time point. Supervised learning was highly effective at determining which participant had taken a given shoe or phone sample, and a Bayesian method was able to determine which participant had taken each shoe sample based entirely on its similarity to the floor samples. Both shoe and phone samples taken by conference participants clustered into distinct groups based on location, though much more so when an unweighted distance metric was used, suggesting sharing of low-abundance microbial taxa between individuals inhabiting the same space.

**Conclusions:**

Correlations between microbial community sources and sinks allow for inference of the interactions between humans and their environment.

**Electronic supplementary material:**

The online version of this article (doi:10.1186/s40168-015-0082-9) contains supplementary material, which is available to authorized users.

## Background

In recent years, research into the microbial interactions between humans and their surroundings has revolutionized our understanding of the microbial ecology of the built environment [1]. The dynamic relationship between the bacteria associated with human skin and the microbiome of indoor surfaces and of objects we interact with has demonstrated the degree to which the human microbiome can shape the microbial ecology of our homes, offices, hospitals, and cities [2-6]. Characterizing this microbial dynamic is critical for many purposes, such as determining the rate and progression of microbial colonization of human infants exposed to the indoor microbiome [7,8]. We therefore believe it is essential to determine how the microbial ecology of the built environment establishes and fluctuates over time.

Research on the microbial exchange between human and built environments has illuminated the forensic potential of the microbiome. In some cases, human microbial signatures have been used to match individuals to objects they have interacted with, including computer keyboards [9]. Work on the microbiome of multiple home surfaces has shown that the microbial signature of a family can be highly predictive of the microbiome of that family’s home and that individuals within a home can be differentiated [2]. Indeed, recent work on the microbial assemblages associated with smart phones has shown that individuals leave their skin microbiome on the surface of their phones [10]. The rate at which these microbial communities change after they are deposited on a surface is also potentially valuable for forensic applications. Recent work has shown that postmortem, the microbiome of animal hosts changes dramatically, but in a predictable manner [11]. This predictability enables us to use microbial assemblages to help explore not just where someone is right now but also where they may have been recently [12]. To explore the potential to determine the microbial fingerprint of individuals on personal items, we performed a detailed biogeographic and longitudinal characterization of the microbial communities on personal mobile phones. Additionally, we examined whether the microbial communities associated with an individual’s shoes were determined by the floor microbiome associated with where they were walking.

## Results and discussion

### Identifying signatures on shoe and phone samples

**Figure 1:**
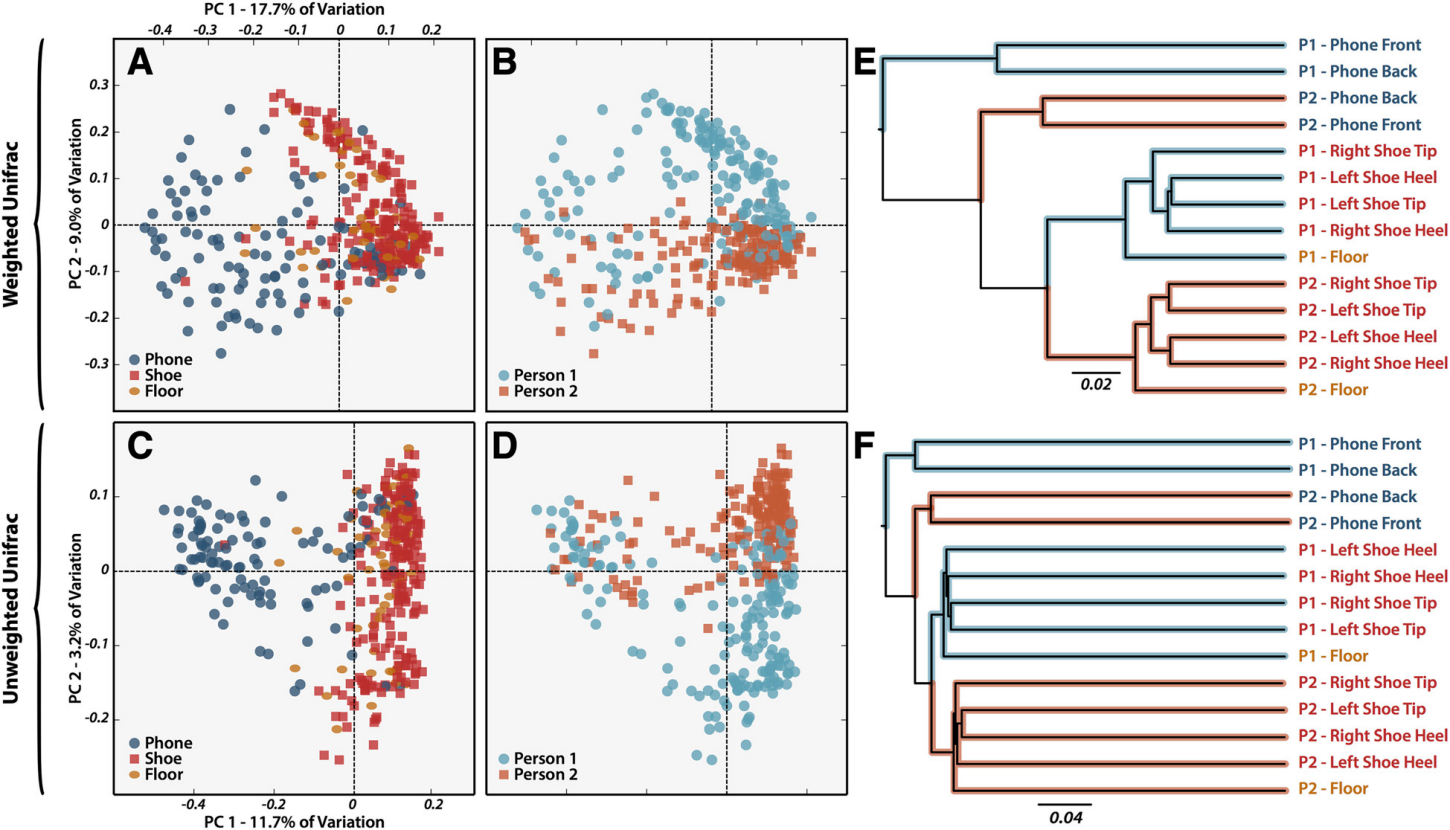
Ordination of samples based on weighted and unweighted phylogenetic dissimilarity in community composition. **(A,** **B)** depict principal coordinate (PCoA) plots for all samples in the study based on pairwise weighted UniFrac distance between samples, with sample points colored by surface and person, respectively. **(C, D)** are similarly colored by surface and person but are based on unweighted UniFrac distance. **(E, F)** depict UPGMA clustering of pooled and evenly rarified sample groupings based on weighted and unweighted UniFrac distance, respectively. Branches are highlighted to reflect person of origin (colors as in B and D) and group names at branch tips are colored by surface as in A and C.

To determine the extent to which the microbial communities of samples were driven by surface type (that is, shoe, phone, or floor) and study participant, we employed a combination of ordination and supervised learning analyses. We found that microbial community structure was determined both by surface type and participant (PERMANOVA on weighted UniFrac; Pseudo-F = 19.7 and 22.7, respectively; *P* < 0.0001). The relative influence of surface type and interacting individual on microbial community structure was demonstrated by the weighted (Figure 1A, B) and unweighted (Figure 1C, D) UniFrac distance between samples. In both cases, the first principle coordinate clearly demarcated sample surface while the second principal coordinate demarcated study participant. UPGMA hierarchical clustering of samples pooled by individual and surface type (Figure 1E, F) further suggested surface type as the dominant influence on microbial community structure, with phone and shoe samples forming distinct groups, which were in turn subdivided individually. In both ordination analyses, floor samples clustered tightly with their longitudinally associated shoe samples.

The diagnostic power of microbial community profiles for predicting which of the two study participants a shoe or phone sample had been taken from was determined using random forest supervised learning. Random forest models were highly successful at determining which of the two participants’ shoes a sample was taken from, correctly classifying samples more than 50 times as effectively as one would expect by chance (Table 1), which indicates consistent differentiation in the shoe microbial communities of these two different people, even accounting for temporal variability. This is likely due to the presence of a ‘core microbiome’ on the shoes of individual study participants, which we assessed by looking at the abundances over time of the 100 taxa with the highest feature importance scores in the model (Additional file 1: Figure S1). The majority of those 100 operational taxonomic units (OTUs) were consistently detected on the shoes of one participant over the course of the time series, but not on those of the other participant.

**Table 1:** **Summary of predictive accuracy of random forest supervised learning models**

**Sample subset**	**Predicted category**	***N***	**Estimated error ± SD**	**Baseline error**	**Ratio**
All phone samples	Person	104	0.037 ± 0.062	0.500	13.63
All shoe samples	Person	211	0.010 ± 0.020	0.479	50.26
P1 phone samples	Front/back	52	0.417 ± 0.206	0.481	1.15
P2 phone samples	Front/back	52	0.268 ± 0.180	0.481	1.79
P1 shoe samples	Shoe surface	110	0.705 ± 0.125	0.736	1.05
P2 shoe samples	Shoe surface	101	0.796 ± 0.090	0.732	0.92

Tenfold cross validation models were constructed with 1,000 trees using OTUs from evenly rarified samples as predictors of sample origin. P1, person 1; P2, person 2, SD, standard deviation, *N*, number.

In contrast to the high error ratio of models predicting study participant, the models did no better than expected by chance in determining which of the four shoe sites a sample had been taken from, even when models were segregated by study participant. We propose that this is due to the homogenization of communities across the shoe sole over time or to rapid changes in community structure at each sampling site. A similar pattern was observed in phone samples, with the models able to classify the participant a phone sample was taken from (error ratio of 13.6) but unable to determine whether the sample had been taken from the front or back of a given phone (Table 1).

Random forest models were also used to assess which bacterial taxa were most associated with different surface types. Models were trained on a genus-level summary of the OTU table, and shoe and floor samples were merged into a single surface type based on their similarity in ordination analyses. When trained at the genus level, models were able to determine whether a sample was taken from a phone or a shoe/floor with an error ratio of 3.6. The 20 genera with the highest feature importance scores are summarized in Additional file 2: Figure S2, with skin-associated genera such as *Streptococcus*, *Propionibacterium*, and *Corynebacterium* highly enriched in phone samples relative to shoe samples.

### Longitudinal interaction between shoe and floor communities

**Figure 2:**
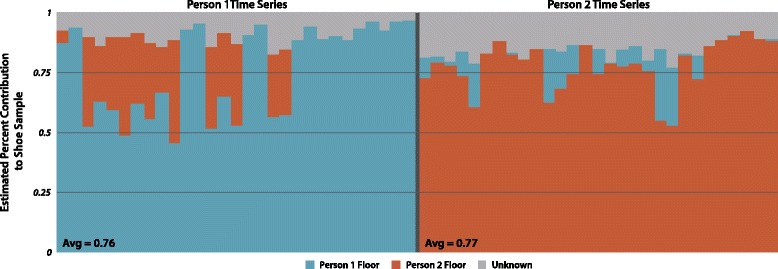
Summary of predictive accuracy of SourceTracker models in determining which of the two study participants a sample was taken from based only on the microbial communities of the floor samples those shoes had interacted with. For the models, all four shoe samples taken by each participant at a given time point were consolidated and treated as individual sinks (*N* = 29 and 27 for persons 1 and 2, respectively). All floor samples from the two participants’ time series were collapsed and treated as the two possible sources to the shoe sink communities.

To determine the extent to which the floor environments a shoe has interacted with influence the sole’s microbial community and to assess whether individual shoe and floor time series could be matched based on similarity, we employed a Bayesian source tracking approach [13]. These Bayesian models predicted a dominant influence from the correct source (Figure 2), which we believe shows the similarity between shoe and floor microbial community composition and may be used to infer where someone has recently walked. On average, the models predicted that a floor sample was the source of microbes for approximately three quarters of the microbial community associated with that shoe at that time point. Strikingly, floor samples had significant predictive power despite often being taken in areas the shoe did not directly touch (that is, proximate to where the participant had actually stepped), which suggests localized homogeneity of the floor microbial community. We also formulated individual SourceTracker models for each participant, in which the floor samples of individual locations were treated as sources to the shoe samples (Additional file 3: Figure S3). These models demonstrated that bacterial taxa associated with the floor of a particular location often increased in abundance on the shoe soles of study participants while walking through that space.

To determine whether changes in the microbial community of the four shoe environments tended to be similar at each hourly sampling interval, we employed Procrustes analysis of the four sets of principal coordinates (Additional file 4: Figure S4). All three pairwise comparisons for each study participant produced significant *P* values (*P* < 0.005; Additional file 5: Table S1), demonstrating that changes in the microbial communities of the four shoe environments resemble each other at each sampling interval, and thus suggesting a consistent impact from the floor microbial community. Procrustes analysis of the principal coordinates from the front and back of participants’ phones did not produce significant *P* values, which we hypothesize is likely due to greater heterogeneity in community composition across the surface area of an individual phone at a given time point than would be observed across a shoe at a given time point due to lower overall biomass and high volatility in hand-associated microbial communities. It is also likely that microbes from the back of phones are likely to be sourced mostly from hands while the front may also be sourced from the face of the owner.

**Figure 3:**
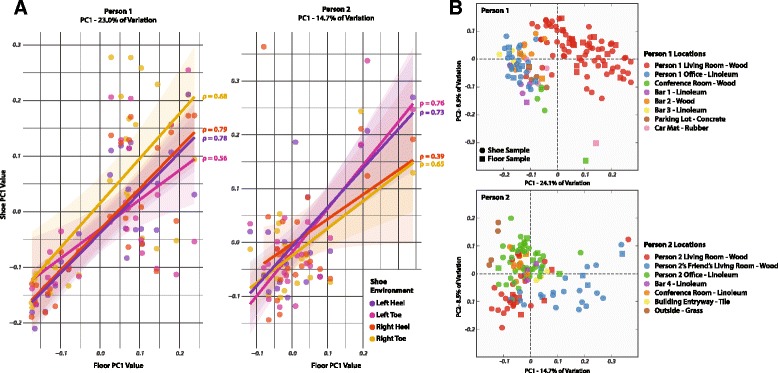
Immediate impact of floor microbial community on shoe microbial communities. **(A)** Correlation in the first principal coordinate values of shoe and floor samples taken at the same time point. **(B)** Principal coordinate plots of all shoe and floor samples, split by individual and colored by floor type and location at time of sampling.

To assess the speed at which the floor environment influences the shoe sole microbial community, we looked at the relationships between shoe and floor samples taken from the same time point in principal coordinate (PC) space. For both study participants, PC1 values for the floor and shoe samples at each time point were highly correlated for all four shoe environments (Figure 3A); we believe this is likely due to rapid contamination of the shoe sole by the floor microbial community. In all but one shoe environment (the right shoe heel of person 2), the correlation between shoe and floor PC1 values from the same time point was substantially higher than the correlation between samples taken one time step apart (Additional file 5: Table S2). In most cases, shoe microbial communities quickly converged on a PC space similar to that of the floor community (Figure 3B). These communities were largely segregated by the geographic location the sample was taken from and by the material of that location’s floor (wood, linoleum, etc.), further supporting the possibility of rapid microbial transfer to the shoe sole.

**Figure 4:**
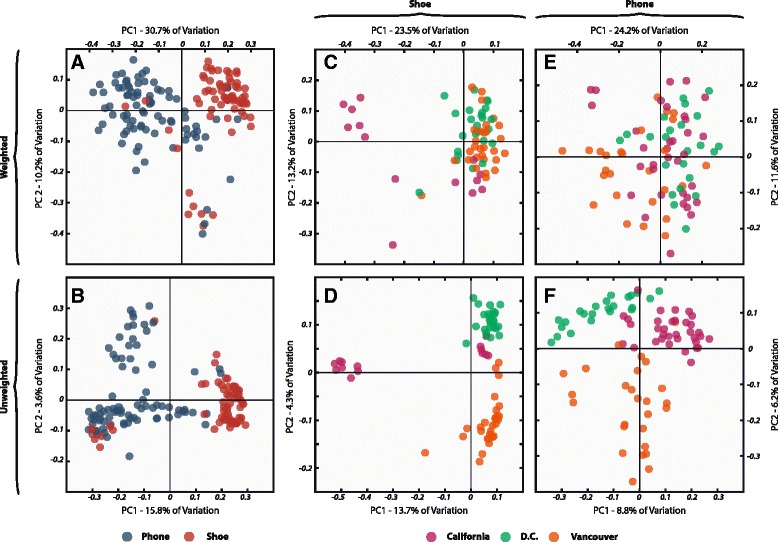
Ordination of biogeographic samples based on weighted and unweighted phylogenetic dissimilarity in community composition. Panels **A** and **B** depict principal coordinate (PCoA) plots for all biogeographic samples based on pairwaise weighted UniFrac distance between samples, with sample points colored by surface and location respectively. **C** and **D** depict ordinations of shoe samples, colored by sampling location, based on weighted and unweighted UniFrac distance, respectively. **E** and **F** depict ordinations of phone samples colored by sampling location and are based on weighted and unweighted UniFrac distance, respectively.

Although our experimental design only allows us to assess the impact of the floor microbial community on that of the shoe sole, it is of course also true that shoes influence floor microbial communities by depositing microbes that have adhered to them. As participants walk, bacteria may adhere to shoes and be subsequently transferred back to the floor in a dynamic process of continual loading and unloading of microbes. A study of uptake and deposit of particles via indoor foot traffic showed that in many cases downplay of particles in the size range of bacteria from shoe to floor is greater than uptake by the shoe [14].

To assess the stability of microbial community structure across the 12 individual shoe and phone time series, we focused on weighted UniFrac distance between samples from consecutive time points and visualized community volatility as a density plot of those distances (Additional file 6: Figure S5). Phone-associated microbial communities were observed to be both less stable (higher median distance) and more variable in their rate of change over time (broader distribution) than shoe-associated communities. By contrast, little difference was observed between the four shoe environments or between the two phone environments. We hypothesize that the high volatility of phone-associated microbial communities is likely due to a small microbial biomass that would be prone to a rapid turnover in community composition and the very high volatility of hand-associated microbiota that has been observed in previous studies [8].

### Biogeographic influence on community structure

In addition to the two time series participants, we also collected individual shoe and phone samples from volunteers at three academic conferences, one in Vancouver, BC (*N* = 29), one in Washington, D.C. (*N* = 26), and one in California (*N* = 34). California samples were taken from two different rooms at the same conference while Vancouver and Washington samples were all taken from the same room. We used these data both to corroborate the patterns of diversity observed in the time series with a larger number of participants and to assess the differentiation in community structure attributable to geographic segregation.

As in the time series analyses, phone and shoe microbial communities were significantly different (Figure 4; Pseudo-F = 38.2 for weighted UniFrac, *P* < 0.0001). The location at which samples were collected also played a significant role in shaping community similarity, especially in shoe samples (Pseudo-F = 8.8, weighted UniFrac, *P* < 0.0001) though also significantly in phone samples (Pseudo-F = 4.9, weighted UniFrac, *P* < 0.0001). Random forest models were able to determine which of the three conferences a sample was taken from significantly better than expected by chance for both the shoe and phone environments (error ratio = 11.7 and 8.0, respectively). This suggests to us that, as seen in the time series data, different sites maintain a significantly different floor microbial community, which in turn shapes the microbial assemblage structure associated with the shoe samples.

## Conclusions

Microbial communities show unique structure and composition based on surface type, the identity of the person interacting with the surface, and geographic location. This has significant implications for a variety of applications. While we suggest that it is possible to infer individual identities based on the microbial community associated with their smart phone surface, it is less likely that this assemblage could be used to track where that person has been recently located in space due to the rapid turnover of the surface-associated microbial community. We believe that the personalized-nature of the human microbiome and the distinct community types associated with urban and built environments may play a significant role in future forensic investigations.

## Methods

### Sample collection

This article reports the results of two studies, one of which employed longitudinal sampling of shoe and phone microbial communities (time series study) and one of which collected individual shoe and phone samples from individuals attending three geographically disparate conferences (biogeographical study). For the time series study, two participants were recruited to sample their shoes and phones every hour over the course of two 12-hour time periods on consecutive days. Samples were collected by the participants by rubbing sterile swabs pre-moistened with 0.15 M saline solution on each site of interest. Floor samples were taken immediately adjacent from wherever the participant was standing at the time of shoe sampling; not necessarily in an area where they had recently stepped. All samples were immediately placed at −20°C, or on dry ice in cases where samples were collected while participants were away from home or office. At each sampling site, participants made note of their current environment and of all actions taken over the proceeding hour. Participant 1 wore flat-bottomed, rubber soled boots while participant 2 wore sneakers with a more complex sole topography. Each participant wore the same pair of shoes on their 2 days of sampling, both with rubber soles.

For the biogeographical study: At three national and international conferences during 2012, samples were collected at random from participants’ phones and shoes. Samples were collected by the participants by rubbing sterile swabs pre-moistened with 0.15 M saline solution on each site of interest. All samples were immediately placed on dry ice and shipped to Argonne National Laboratory, where they were stored at −80°C until processed.

### Library preparation

Total DNA was extracted from swabs using the Extract-N-Amp plant PCR kit (Sigma, St. Louis, USA) following the manufacture’s protocol with minor modifications. After extraction, DNA was quantified using PicoGreen (Invitrogen, Grand Island, USA) and a plate reader. DNA was then amplified using the Earth Microbiome Project barcoded primer set, adapted for the Illumina HiSeq2000 and MiSeq (Illumina, San Diego, USA) by adding nine extra bases in the adapter region of the forward amplification primer that support paired-end sequencing. The V4 region of the 16S rRNA gene (515 F-806R) was amplified with region-specific primers that included the Illumina flowcell adapter sequences and a 12-base barcode sequence [15,16]. Each 25 μl PCR reaction contained the following: 12 μl of MoBio PCR Water (Certified DNA-Free; MoBio, Carlsbad, USA), 10 μl of 5 Prime HotMasterMix (1×), 1 μl of forward primer (5 μM concentration, 200 pM final), 1 μl of Golay Barcode Tagged Reverse Primer (5 μM concentration, 200 pM final), and 1 μl of template DNA. The conditions for PCR were as follows: 94°C for 3 min to denature the DNA, with 35 cycles at 94°C for 45 s, 50°C for 60 s, and 72°C for 90 s, with a final extension of 10 min at 72°C to ensure complete amplification. Amplicons were quantified using PicoGreen (Invitrogen) and a plate reader. Once quantified, different volumes of each of the products are pooled into a single tube so that each amplicon is represented equally. This pool is then cleaned up using UltraClean® PCR Clean-Up Kit (MoBio, Carlsbad, USA), and then quantified using Qubit (Invitrogen, Grand Island, USA). After quantification, the molarity of the pool is determined and diluted down to 2 nM, denatured, and then diluted to a final concentration of 4 pM with a 30% PhiX spike for loading on the Illumina HiSeq2000 sequencer (for the time series study), and a final concentration of 6.1 pM with a 30% PhiX spike for sequencing on the Illumina MiSeq (for the biogeographical study).

### Sequence processing and analysis

Unpaired reads of length 151 bp for both the time series and biogeographic studies were clustered together at 97% identity using the Quantitative Insights Into Microbial Ecology (QIIME) script *pick_open_reference_otus.py*, with the May 2013 release of Greengenes (greengenes.lbl.gov) as the reference. OTUs comprising only a single read were discarded, and samples were rarified to an even depth of 1,000 reads.

Analysis of beta-diversity was performed by calculating the pairwise weighted and unweighted UniFrac [17] distance between each pair of samples, and the resulting distance matrix was used for all downstream statistical tests of sample similarity. The significance of sample groupings was assessed using PERMANOVA (QIIME’s *compare_categories.py* script) and statistical significance was calculated by comparing the Pseudo-F statistic to a distribution generated by 10,000 permutations of the randomized dataset.

### Random forest models

Random forest supervised learning models were used to determine the diagnostic power of microbial community profiles in predicting the surface type or participant a sample originated from. These models form decision trees using a subset of samples to identify patterns associated with a metadata category and then test the accuracy of the tree on the remaining samples not used for training. Each model runs a number of independent trees and reports the ratio of model error to random error as a metric for the predictive power of the category’s microbial communities. A greater ratio of baseline to model error indicates a better ability to classify that grouping by microbial community alone. The models were run using the *supervised_learning.py* command in QIIME, with 1,000 trees per model and tenfold cross validation.

### SourceTracker models

For the SourceTracker models, all four shoe samples taken by each participant at a given time point were consolidated and treated as individual sinks (*N* = 29 and 27 for participants 1 and 2, respectively). All floor samples from the two participants’ time series were collapsed and treated as the two possible sources to the shoe sink community. Models were run following QIIME tutorial guidelines (http://qiime.org/tutorials/source_tracking.html).

### Procrustes

Procrustes analysis compares the shape of two PCoA plots by optimally rotating and scaling one plot to best fit the other, with the goodness of fit measured by the *M*2 statistic. *P* values are generated using a Monte Carlo simulation in which sample identifiers are shuffled (here 1,000 times) and the *M*2 statistic is compared to the distribution drawn from these permutations. The proportion of *M*2 values that are equal or lower than the actual *M*2 value is the Monte Carlo *P* value.

Only time points in which all four shoe samples passed quality filtering were considered (*N* = 24 for participant 1 and 19 for participant 2). For each participant, samples were divided by shoe environment and four different sets of principal coordinates were computed based on weighted UniFrac distance between samples. The QIIME script *transform_coordinate_matricies.py* was used for Procrustes analysis, with the left heel coordinates used as the reference and the other three coordinate matrices transformed to best fit the reference.

### Availability of supporting data

All sequencing data as well as the OTU table and mapping file are available at http://figshare.com/articles/Forensic_analysis_of_the_microbiome_of_phones_and_shoes/1311743.

## Additional files

Additional file 1: Figure S1. Heatmap of the abundances of the 100 OTUs with the highest feature importance scores in the random forest model differentiating shoe samples by participant. Each row represents a single OTU. All shoe samples taken by a participant at each time point are collapsed, and blocks are ordered first by participant and then by time. Additional file 2: Figure S2. Heatmap of the abundances of the 20 genera with the highest feature importance scores in the random forest model differentiating shoe/floor and phone samples. For the heatmap, all samples taken from a given surface environment were collapsed across time points and heatmap color was normalized for each genus. Additional file 3: Figure S3. SourceTracker models for individual participants. All floor samples taken at each location were consolidated and treated as possible sources. All four shoe samples per time point were consolidated and treated as sinks. Bar height represents the mixing proportion estimate for each source in each sink sample, with the source environment where the participant was located at time of sampling indicated by a higher opacity and a black box. For person 2, time point 10, the participant was on lawn outdoors and the floor sample failed to produce enough reads to be included in the study. Additional file 4: Figure S4. Procrustes analysis of shoe samples, demonstrating relatedness of community succession in the four shoe environments sampled in each person’s time series. Samples in the PCoA plots are colored by the time point in which they were taken, and the four samples per time point (left heel, right heel, left tip, right tip) are connected by edges. Additional file 5: Table S1. Summary of goodness of fit and respective Monte Carlo *P* values for each Procrustes alignment in Figure S4. **Table S2.** Correlation between PC1 values for floor and shoe samples taken from the same time point (as in Figure 3A) and with a plus or minus 1 time point differential. In all but one case, correlation is highest with no time lag, suggesting an immediate impact of the floor community on the shoe communities. Additional file 6: Figure S5. Volatility of individual surfaces across their time series, visualized as density plots of weighted UniFrac distances between samples from consecutive time points. Plots are colored by the median distance in those series of consecutive distances.
