# Supplementary figures and images for: Forensic analysis of the microbiome of phones and shoes
Source: Microbiome. 2015 May 12;3:21. doi: 10.1186/s40168-015-0082-9 (PMC4427962; doi:10.1186/s40168-015-0082-9)

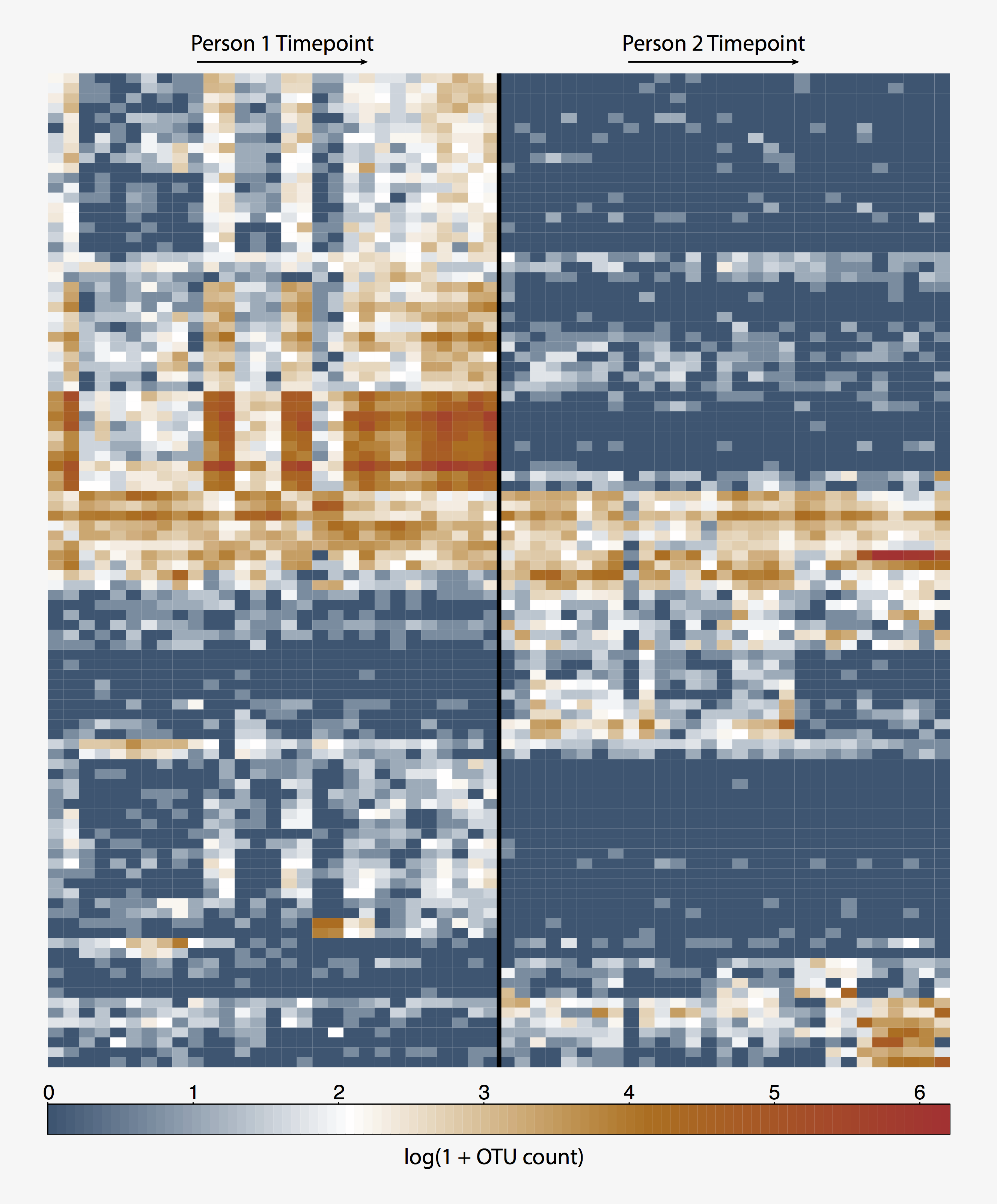

Supplement: Additional file 1: Figure S1. — Heatmap of the abundances of the 100 OTUs with the highest feature importance scores in the random forest model differentiating shoe samples by participant. Each row represents a single OTU. All shoe samples taken by a participant at each time point are collapsed, and blocks are ordered first by participant and then by time. [file 40168_2015_82_MOESM1_ESM.png]

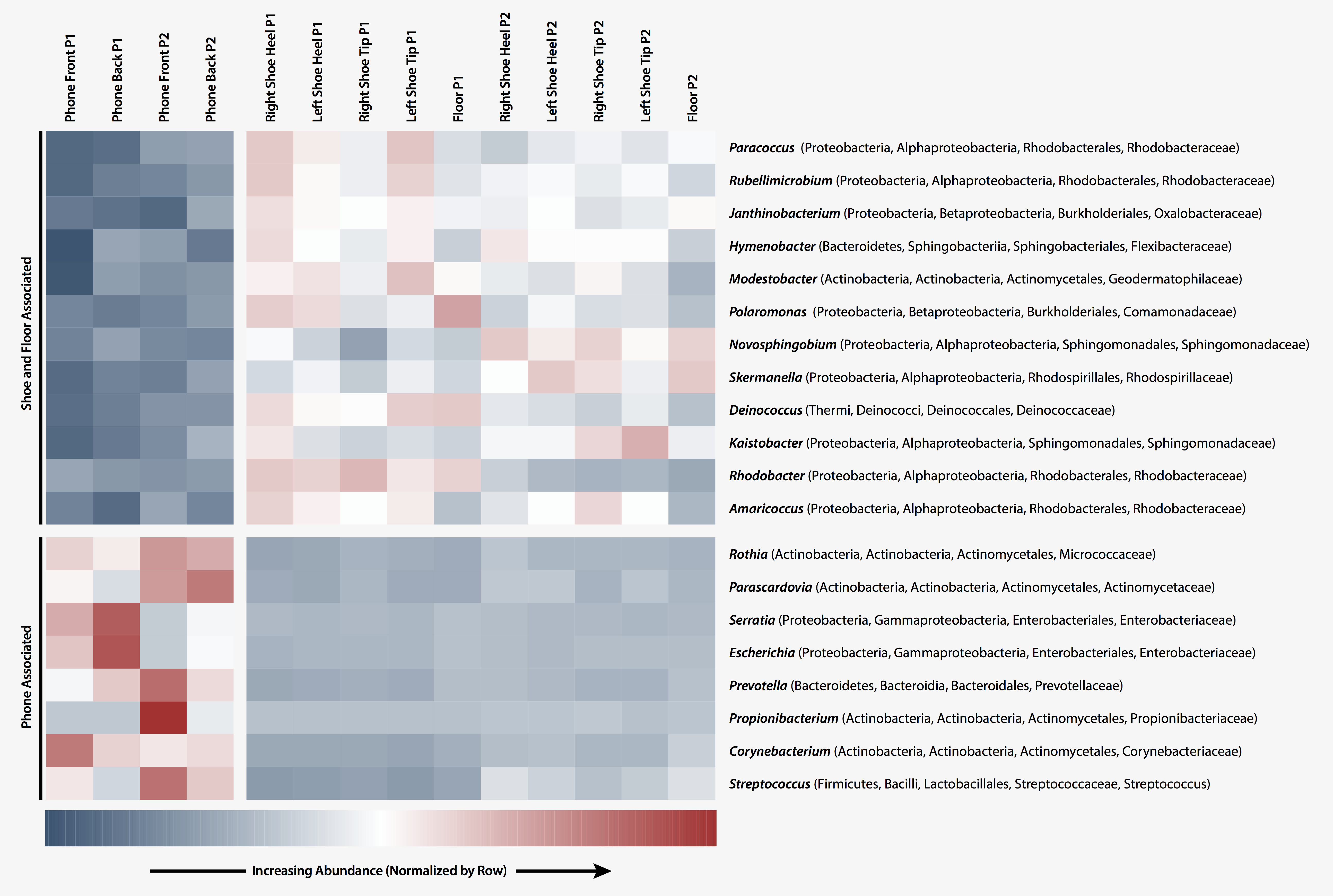

Supplement: Additional file 2: Figure S2. — Heatmap of the abundances of the 20 genera with the highest feature importance scores in the random forest model differentiating shoe/floor and phone samples. For the heatmap, all samples taken from a given surface environment were collapsed across time points and heatmap color was normalized for each genus. [file 40168_2015_82_MOESM2_ESM.png]

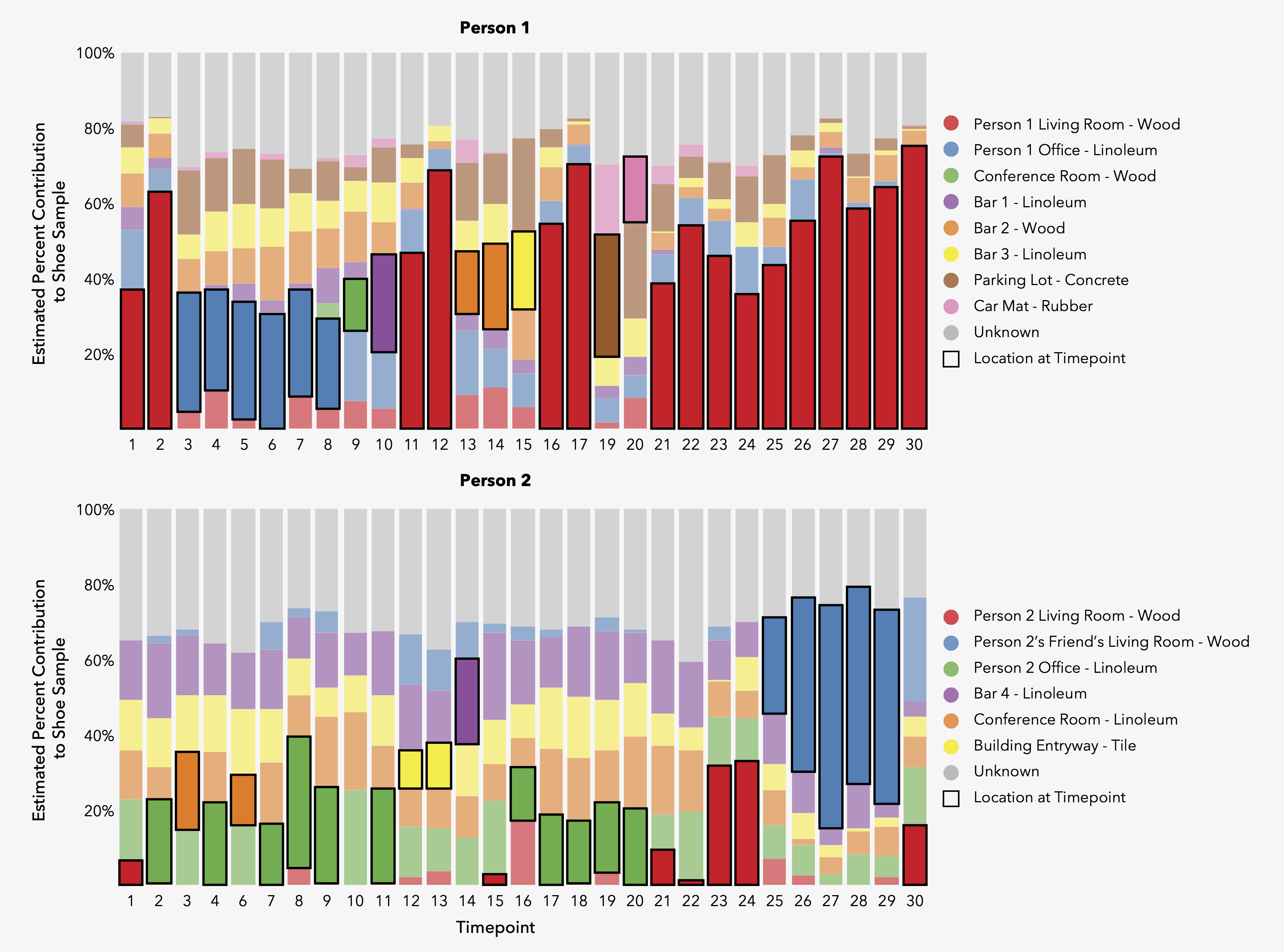

Supplement: Additional file 3: Figure S3. — SourceTracker models for individual participants. All floor samples taken at each location were consolidated and treated as possible sources. All four shoe samples per time point were consolidated and treated as sinks. Bar height represents the mixing proportion estimate for each source in each sink sample, with the source environment where the participant was located at time of sampling indicated by a higher opacity and a black box. For person 2, time point 10, the participant was on lawn outdoors and the floor sample failed to produce enough reads to be included in the study. [file 40168_2015_82_MOESM3_ESM.png]

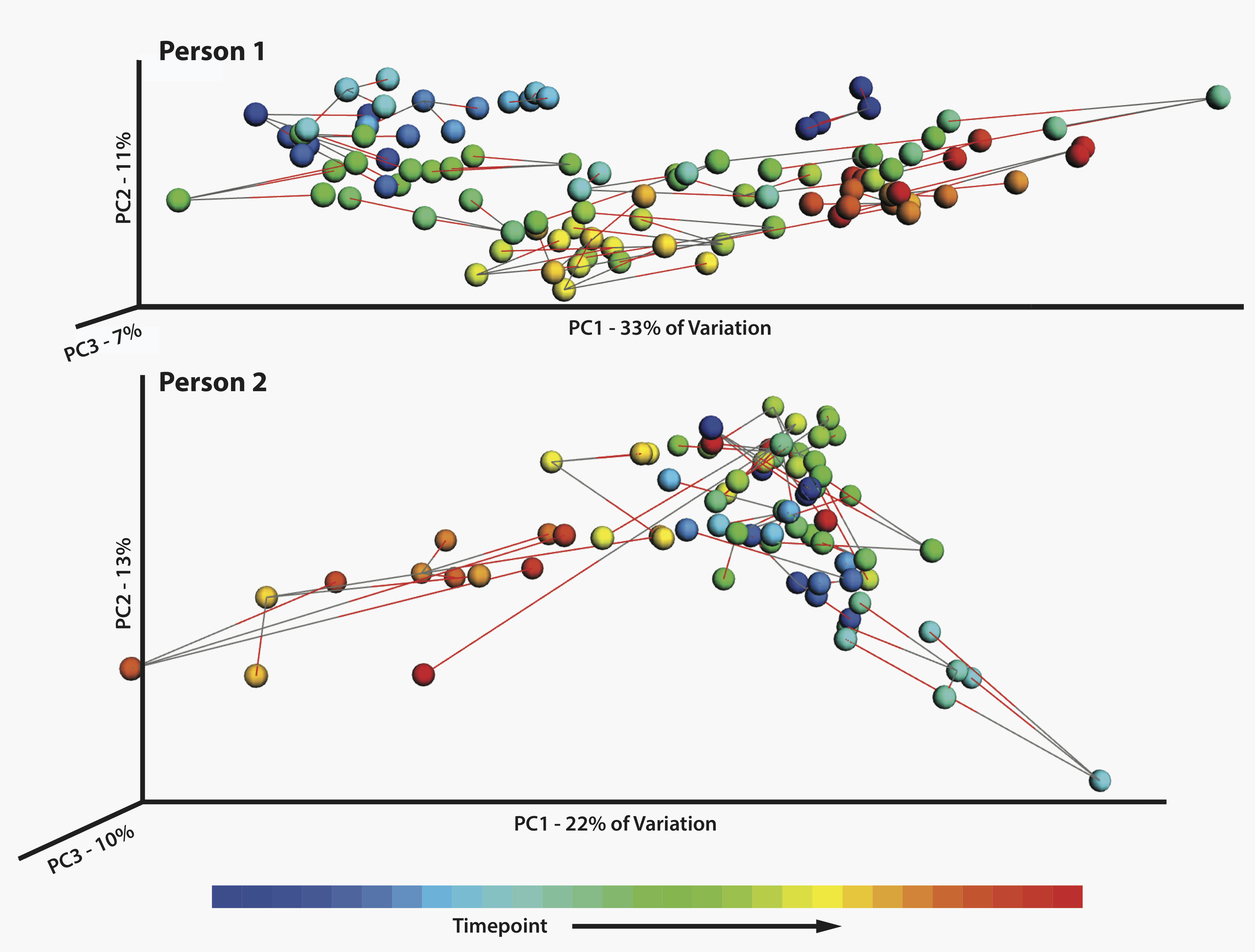

Supplement: Additional file 4: Figure S4. — Procrustes analysis of shoe samples, demonstrating relatedness of community succession in the four shoe environments sampled in each person’s time series. Samples in the PCoA plots are colored by the time point in which they were taken, and the four samples per time point (left heel, right heel, left tip, right tip) are connected by edges. [file 40168_2015_82_MOESM4_ESM.png]

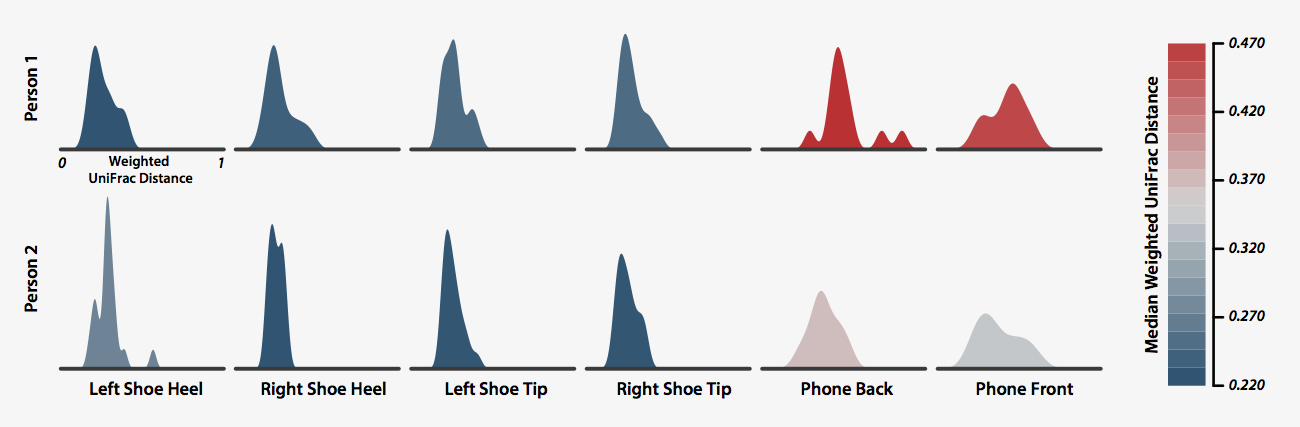

Supplement: Additional file 6: Figure S5. — Volatility of individual surfaces across their time series, visualized as density plots of weighted UniFrac distances between samples from consecutive time points. Plots are colored by the median distance in those series of consecutive distances. [file 40168_2015_82_MOESM6_ESM.png]
